# Cyberchondria Amidst COVID-19 Pandemic: Challenges and Management Strategies

**DOI:** 10.3389/fpsyt.2021.618508

**Published:** 2021-04-30

**Authors:** Rahul Varma, Sreeja Das, Tushar Singh

**Affiliations:** Department of Psychology, Banaras Hindu University, Varanasi, India

**Keywords:** cyberchondria, COVID-19, health, health related internet searches, Covid anxiety

## Abstract

The corona-virus disease 2019 (COVID-19), first found in Wuhan, China in December 2019, has posed an inexplicable threat to the global community. After its inception, the virus proliferated rapidly, which led to the cause of millions of deaths, and having a detrimental effect on physical health, social lives, economic uncertainty, and mental health of people. The World Health Organization has reported that there are 111 million confirmed cases of COVID-19 and 2.45 million deaths due to COVID-19 worldwide. Indisputably, the present pandemic has contributed to the extensive psychological and environmental distress together with clinical depression, anxiety and post-traumatic stress disorder (PTSD), domestic violence, and unemployment. Due to the ambiguous nature of the pandemic, educational organizations, and outdoor activities are closed, thus burdening the mental health of younger populations. Children as well as youths are more glued to the Internet for their studies, online gaming, shopping, watching movies, and searching health-related information. Despite the advantages of using the Internet, it has some severe consequences too. Some people are repeatedly searching for physical and mental well-being related information without verifying credible sources, which, in turn, causes distress and anxiety. In such situations, individuals may end up contributing to an illness known as cyberchondria. In this paper, we have tried to highlight the problematic use of Internet for health-related searches and have outlined the management of such illness. We suggest two strategies: firstly, to reduce repeated online searches of health information and, secondly, to manage anxiety-augmenting thoughts that are triggered due to the maladaptive thoughts caused by the abstruse information.

## Introduction

The global trudge of COVID-19 is beginning to look inexorable. The WHO reported cases of pneumonia due to an unknown cause in the Wuhan city of China on December 31, 2019. On further probing, Chinese authorities identified the novel virus as coronavirus on 7th January and was provisionally named as “2019-nCoV.” As the year 2020 progressed, numerous cases of the novel coronavirus proliferated in most cities of China, and due to its highly permeable nature, the virus transmitted rapidly to other countries; therefore, the WHO declared it as a pandemic on March 11, 2020. As of February 22, 2021, there have been 111,114,777 confirmed cases of COVID-19 and 2,461,436 deaths (1) due to this pandemic. In the interim, recent research projects have focused on new symptoms, diagnosis, management, and development of vaccine and drugs (2).

Pulla (3) reported that India observed its first COVID-19 positive patient on January 31 and the nationwide lockdown in India was initiated from March 24, 2020 and was extended until May 31, 2020. Although the unlocking phase was initiated in June 2020, many schools, colleges, and universities are still not functional. This extended quarantine period and the control measures associated with COVID-19 have their enormous effects on masses (4, 5). The Indian Express (6) outlined the guidelines of unlock of 5.0 by stating that the Indian government has allowed to open schools, theaters, and swimming pools in many states of the country in mid-October while maintaining social distancing norms, wearing masks, and thermal scanning at every entry point. In India, it was the first time that such a restrictive course of action was taken to restrain the contamination. These measures, thus, have greatly affected the lifestyle (e.g., education, working, and social interactions) of the people.

Recent reviews propounded that the psychological repercussions of social distancing and isolation are substantial, are broad ranging, and can be enduring, comprising mood disorder and anxiety, PTSD and psychological distress, and other psychopathological conditions (7, 8). Some studies have revealed that availing the Internet and social networking forums for reduction of stress, fear of illness, and anxiety has elevated amid the COVID-19 pandemic, and for individuals, problematic Internet use (PIU) may fall along with the reduction of stress and anxiety (9, 10). Searching health information on the Internet can also be problematic; if individuals are using the Internet as a diagnostic tool for their illness, with less or no medical literacy, it will probably heighten their anxiety (11).

People diagnosed with hypochondriasis are prone to look for medical information because of their fear of illness (12, 13). When individuals with their somatic symptoms, health anxiety, and distress use social media and the Internet to get the information associated with their health, they are embodied as having cyberchondria (14). Cyberchondria has not been included so far as a distinct diagnosis in the Diagnostic and Statistical Manual of Mental Disorders (DSM-5) (15), but it is a kind of anxiety disorder in which individuals conduct an Internet search and, based on the search result, they conclude that they have an illness (16). Mostly, they discern the illness in perilous form, which shoots up their anxiety and fear (14).

## Psychosocial Impact of Covid-19 Pandemic

The present pandemic has globally impacted not only the health but also the economic status of individuals (17). The pandemic and the resulted lockdowns imposed by the Governments to stop the spread of the disease have resulted in various psychological issues including, but not limited to, clinical depression, anxiety, post-traumatic stress disorder (PTSD), Suicidal ideation and suicide (18), domestic violence (19, 20), Stigma, discrimination (21) and unemployment. Not only the general population but also the frontline healthcare professionals have been reported to experience psychological distress, anxiety, depression, delusion, Suicidal thoughts and death (22–24). Besides, the global health distress, the COVID-19 pandemic has a detrimental upshot on the world economy as well, which has resulted in the depreciation in the overall GDP (25). The inescapable nature of the pandemic has malformed the Indian economy leaving the country shattered and directionless (26). Chaudhary et al. (26) highlighted the catastrophic condition of daily wagers, migrant workers, and MSMEs (micro, small, and medium enterprises), which resulted in a major threat to the economy of the country. In the current situation, after 9 months of being unlocked, the MSMEs are on a spree of opening shops in the most vulnerable locations to meet their basic needs.

With the continuous rise of the infection, there is an increasing rate of health-related issues among people (27). Shadmi et al. (28) reported that frontline professionals and workers, who are comparatively more exposed to public, are more vulnerable to this infection. They also stated that the people who belong to lower economic strata and migrant workers are more susceptible to infection and fail to seek help due to unavailability of finance and poor access to healthcare facilities. This leads to poor prognosis, which may result into death. According to Arumugam et al. (29), patients with comorbid diseases such as hypertension, diabetes, and heart disease have a higher mortality rate. Adding to the problems, due to the infectious nature of the pandemic, some necessary treatments and surgeries have been delayed for patients who are diagnosed with cancer or other major illnesses (30) and the delay of such surgeries for tumors has resulted in the advancement of the tumors from treatable to untreatable (31). According to The Lancet Rheumatology (32), there are also delays in elective surgery, i.e., surgery that people choose to have a better quality of life but not for a life-threatening condition, e.g., hernia surgery, cataract surgery, cardiovascular surgery, etc., and also some orthopedic surgeries such as for osteoarthritis. These diseases often cause debilitating discomfort that interrupts mobility and obstructs with daily routine. Living with chronic pain induced by an illness or a disease may result in substance abuse and impaired mental health.

The pandemic has stemmed to limit face-to-face contact. Zero physical contact has led to disrupted social lives contributing to antagonistic psychological upshots including loneliness, clinical depression, trauma, domestic violence, and health anxiety (33). Adverse cases of obsessive-compulsive disorder are also observed, which are caused by decreased belief in healthcare structure and people are donned with fear of contracting the infection (34). Recent studies have also suggested prevalent symptoms of PTSD also due to the aftermath of this current pandemic (35).

It can, therefore, be concluded that the current COVID-19 pandemic is giving considerable rise to physical and psychological stress and high morbidity and mortality rates all over the world since its upsurge in December 2019 (36, 37). Jalloh et al. (38) found in their research that up to 50% of the respondents in their studies reported anxiety or worries during virus-induced pandemics or epidemics. Also, in a few recent studies conducted in China among general population and adults, it was found that about 25–35% of respondents experienced psychological stress or anxiety symptoms during the COVID-19 pandemic (36, 39).

## Cyberchondria Linked to Psychological Health, Anxiety, and Stress

The Internet has crawled into people's lives and has gradually become an umbilical to the peripheral world. Individuals are dependent on Internet connection for majority of reasons as it has replaced schools, jobs, and face-to-face communication with family and friends. Although online health-related information search has some latent benefits that help to enlighten people about ailments, their remedies and treatment (11), some people are repeatedly searching physical and mental well-being-related information to quench their thirst of queries, which, in turn, causes distress and anxiety (40).

The abnormal practice of searching health information on the Internet to alleviate stress and anxiety but instead worsening the condition is called cyberchondria (41). It refers to the unfounded increase in concerns about general symptomatology, which is based on Internet search results (11). Cyberchondria has been inextricably linked to escalated health anxiety, stress, and depression and is also associated with obsessive-compulsive disorder (OCD) (14, 42–44). Sarkar (45) reported that the detrimental effect is mainly pictured in the youth population, which is techno geek. He further added that cyberchondria elevates distress consecutively causing high blood pressure, anxiety, and muscle spasm, which are generally triggered by an event like a sick person or the news of a death of someone close to them.

In the present world, the World Wide Web is the source for almost every piece of information for most people. Many of us access the Internet on a daily basis to get different kinds of information from it. And now we have also started using also the Internet to get health-related information. There are many websites available on the internet that can give us misleading information about health-related conditions and this can escalate anxiety and stress. Self-diagnosis and self-treatment may put people at risk as they have less or no medical knowledge and do not have descriptions for the medical conditions. These factors cumulatively make searching the Internet for health-related information more misleading and dangerous (46).

A recent study conducted in Oman by Al Dameery et al. (47) shows that there is a strong correlation between cyberchondriac experience and psychological stress. In their meta-analytic study, McMullan et al. (15) have presented a significant relationship between cyberchondria and health anxiety and have demonstrated the commonality between the two constructs. Using a structural equation modeling approach, Fergus and Russell (48) found that while cyberchondria overlaps with the affective (health worry) and perceptual components (increased vigilance for physical symptoms) of health anxiety, it does not relate to its cognitive (dysfunctional health beliefs) and behavioral components (avoidance or reassurance seeking). These results, together with other studies (49, 50), suggest that cyberchondria is an overlapping, yet distinct, entity in relation to health anxiety.

## Prevalence of Cyberchondria During Covid-19

As per the Internet World Stats (51) data ~4.93 billion people worldwide are Internet users (September 2020), and most of its users are substantially located in Asia (51.8%) followed by Europe (14.8%) and Africa (12.8%). North America has the greatest Internet penetration rate (% of population using the Internet) at 89.9%, with Europe at 87.1%. The world average Internet penetration rate is 63.2%, indicating that the Internet has become the established medium for the dissemination of targeted messages to a huge audience (51). The Internet has become an alternative for a health practitioner, as outlined in a survey study conducted across 12 countries, where more than 12,000 individuals participated and showed that nearly half of them used “Google” as a search engine for self-diagnosis (52). The Telegraph (An Indian English daily newspaper) in March 2019 quoted the vice president and MD, of Google Health, saying that ~7% of daily Google's searches belong to health-related searches, which account for about 70,000 searches per minute.

Due to the stay-at-home order by the governments during the COVID-19 pandemic, institutional organizations are closed, and people are asked to work from home. As a result, people's daily lives are being governed by the Internet like never before (53). Additionally, due to online classes, and work from home arrangements people are spending much more time on social media and playing online video games (54). When we compare the COVID-19 pandemic from previous large-scale epidemics, we get one novel issue related to mental health and this is the increased problematic use of the Internet (9). This may be because of the prolonged period of home quarantine and restrictions on face-to-face contact; because of which, people may undergo through greater distress and seek an escape through online activities (55). People's insecurity and anxiety for the disease can push them toward compulsive checking for information online which further escalate their anxiety, creating a vicious cycle of cyberchondria that is hard to stop (56, 57).

People perform all these health-related searches to reduce their stress and anxiety about the COVID-19 pandemic, but it may develop into habits of Internet searching and surfing that are difficult to break (58). The Internet and social media are flooded with information related to COVID-19. In news and articles, most of the pieces of information were discovered to be incomplete and inaccurate (59). Doherty-Torstrick et al. (60) found that truckloads of news information obscurely bundled with the curiosity on the epidemic situation heightened the health anxiety.

The novel case of COVID-19 came up rapidly, and this developed phobia among individuals. There are news and information all over the Internet and social media, and people started spending more and more time to collect information about it. These information, however, are not always authentic. Sometimes they are from some reliable sources but for most of the times they are only rumors and/or are based on false/misleading information/sources. This further adds to confusion in recognizing actual circumstances.

When these pieces of information are being produced and transferred speedily, most of the information is not put together and introduced in an optimal and perspicuous way. It creates vagueness among people, and it contributes to cognitive overload, which could be corroborated from previous studies which suggested that cyberchondria is correlated with cognitive overload (11) and uncertainty (61). Also, in a latest research Laato et al. (62), found that cyberchondria is a side effect of the COVID-19 pandemic. The main reason mentioned by them is increased trust on online content, which leads to sharing unverified information. Laato et al. (62) identified a positive correlation of cyberchondria with four major factors: reliance on the information a person is getting from online resources, information overload, perceived severity, and perceived vulnerability. It is, thus, the need of the hour to manage the implications of cyberchondria as the world is facing a global pandemic.

## Management Implications

Due to the exponential increase in the role of the Internet in today's world, it is impossible to cut down on online searches of health-related information. Henceforth, it is imperative to manage and monitor the content of online searches. Though the treatment of cyberchondria is in its post-natal stage, researchers have developed a tool for its diagnosis, and little analytic attention has been paid in regard to its treatment. Cyberchondria has been included neither in the Diagnostic and Statistical Manual of Mental Disorders (DSM-5) nor in the International Statistical Classification of Diseases and Related Health Problems (ICD-11). This article builds on and contributes to discussion about the two aspects of treatment: the first aspect is to repeatedly curtail online health-related searches, and the second aspect is to manage anxiety-amplifying thoughts that arise due to the distorted cognition caused by ambiguous information. Starcevi and Berle (16) claimed that a person might primarily adhere to medical sites or forums that are credible and reliable, and then can eventually shift to critically appraising the content of the information. Instead of imposing the opinion to isolate oneself from the internet, one should stay online in a controlled fashion to avoid threatening and alarming information and a regular check-up with a doctor is a must for someone who notices any kind of abnormality in his or her body.

Due to the upsurge of the Internet and its accessibility, the youth is blindly following online information without thinking of its integrity. Henceforth, family members should educate their wards to be aware of the reliability and validity of the information source. This could be corroborated with the existing findings of Starcevi and Berle (16), who emphasized that, irrespective of the underlying factors, psycho-education is indispensable to improve online health reliant information literacy.

Psycho-education can play a significant role in reducing the effect of cyberchondria. Once the symptoms of cyberchondria have been diagnosed, it is essential to inform the patients about its detrimental effects and all the possible outcomes. We can make them aware of the negative consequences of their problematic use of the Internet for health purpose. Educational policies should be designed to advice patients about the credibility of online health searches, understand the information, and then incorporate it into their lives to manage their health problems (16, 42).

The increase in the online search of health-related information could be due to expensive doctor's visiting fees and treatment. Also, mistrust could also be a reason to avoid visiting doctors (63). Therefore, it is advisable for doctors to form a rapport with their patients and lend them an ear to understand their thought process and belief system, and clarify their doubts.

Metacognitive beliefs, particularly about the uncontrollability of thoughts, appear more relevant to cyberchondria (64) metacognitive treatment strategies, thus, become an important part of it's treatment package. The treatment helps in restructuring negative metacognitive beliefs. For instance, individuals may indulge themselves in detached mindfulness which is a novel metacognitive technique that focuses on memory, increases metacognitive awareness, and detaches oneself from predisposed thinking (65). Further the engagement phase, mainly focus on attentional modification and challenging metacognitive beliefs with respect to Internet use. During this phase, one may indulge in situational attentional refocusing, which impedes the patterns of set attention, maintains perceptions that are menacing, and enables the inconsistent metacognitive beliefs. Spada (66) sketched that individuals may cultivate the skill to purposefully guide their attention to non-verbal signals so as to stop themselves from indulging in repeated online searching behavior for health-related information.

At this point of time when the world is facing a global pandemic, with the sudden restrictions and limitations, it is problematic to visit a doctor every now and thus, one should focus on e-counseling from certified counselors or psychologists. Newby and McElroy (67) found in their study that people have experienced improvement in treating cyberchondria after getting the Internet-based Cognitive Behavior Therapy (iCBT). They found that following iCBT, there were major improvements on distress, compulsion, and excessiveness subscales of cyberchondria and moderate improvements on reassurance subscales. The result of this study suggests that the iCBT may help to reduce the repetitive behavior of online search of health information, the distress it caused, and lessen the effect of online searching on daily activities. The iCBT may also motivate people to consult with a health professional or an expert of that area to seek reassurance.

DoctorxDentist is a Singapore-based online medical portal that offers a convenient and easy way of getting information about COVID-19, and it also helps people avoid fake information. The DoctorxDentist platform provides specialist doctors and experts from the medical field, and these doctors and experts are well aware of the causes and consequences of cyberchondria. In this pandemic situation, the platform is providing articles related to COVID-19 for free to people who are searching for them. There is a team of doctors available for any kind of questions and queries. An individual may contact them for any query s/he has and the same would be answered by an expert doctor from the team. The same doctor can also be approached for an appointment if the individual develops any symptom related to any illness. It, therefore, is also a good initiative to prevent people from getting fake information online and also keep them away from information overload.

Furthermore, with the existing scenario of COVID-19, zero contact with the outer world and constant news of mishap compel one to stay active and worry about one's health. Thus, it causes citizens to neglect the above-mentioned activities to alleviate anxiety-intensifying thoughts. Consequently, it is cardinal for family members, if staying with patients, to closely monitor and regulate their daily activities. Parents should spend more time with their children and participate in fun activities, for example, scrabble, monopoly, painting, or dancing, thus creating a healthy environment. This would increase coping skills and create a stronger support network for priority groups. Anxiety-provoking thoughts could be further alleviated with the practice of relaxation techniques, yoga, and mindfulness.

## Conclusion

The world has come to a standstill due to the COVID-19 pandemic. It has left a trail of destruction that is unprecedented in recent public memory, with 111,114,777 cases and global death surpassing 2,440,000 reported to date (1). Such epidemic took a toll on the mental health of the citizens. Adjusting to new lifestyle challenges, for example, working from home, attending online classes, and no contact with the outside world, has become very challenging for the entire world. In the existing scenario, indulging in maladaptive activities has also become logical. Citizens, particularly the youth, who have been constantly glued to the media, are indulging themselves in health-related online searches. The repetitive health-related online searches from unreliable sources have led to the maximization of anxiety-provoking symptoms called “Cyberchondria” which is a form of excessive health-related online searches by people who are extremely concerned and anxious about their health, which often results into a perplexed state of mind.

Although many researchers are working on cyberchondria, a separate manual for its treatment remains unexplored and unexamined. It is advisable to include cyberchondria as a disorder in one of the diagnostic manuals as such symptoms are often observed across the globe. In the current situation, citizens are often afraid to visit a doctor. Therefore, it is suitable to opt for e-counseling, which is provided at no cost or with minimal charges keeping the pandemic in mind. The counselors should focus on uploading online materials to educate the priority group to understand more about their behaviors and thoughts in an adaptive fashion. One should also be trained to differentiate between reliable and non-reliable sources and to try techniques such as mindfulness, meditation, yoga, and relaxation to calm oneself. Parents are also recommended to spend time with their children, monitor their day-to-day behavior, and also decrease their screen time. Finally, with the increase in digitalization, the decrease in Internet usage is not plausible; hence, it becomes vital for people with cyberchondria to be able to use the Internet in a controlled manner.

## Limitations and Further Suggestions

The present article attempts to highlight the theoretical perspective of cyberchondria and its upsurge during the pandemic. However, this article is not devoid of limitations. Despite the article discussing about the possible strategies to prevent cyberchondria, it does not provide any empirical evidence in its support. Thus, future studies should focus on the planning and administration of these strategies to evaluate their effectiveness. Also the present paper did not put emphasis on susceptible factors causing cyberchondria, for example, hereditary factors and dispositional factors. Therefore, future studies are needed to explore the factors, other than repeated online health searches, which can lead to cyberchondria. The future studies should also put emphasis on the relation between cyberchondria and other forms of PIU and psychopathological underlying morbidities (14).

## Data Availability Statement

The original contributions presented in the study are included in the article/supplementary material, further inquiries can be directed to the corresponding author/s.

## Author Contributions

RV, SD, and TS conceptualized the outline of the manuscript. RV and SD prepared the first draft and final version of the manuscript. TS reviewed the manuscript and provided critical observations. All authors contributed to the article and approved the submitted version.

## Conflict of Interest

The authors declare that the research was conducted in the absence of any commercial or financial relationships that could be construed as a potential conflict of interest.
